# Properties and Characteristics of Film from Salmon Skin Acid-Soluble Collagen Solution as Influenced by Ultrasonication Process

**DOI:** 10.3390/foods14071088

**Published:** 2025-03-21

**Authors:** Krisana Nilsuwan, Sujinun Thongnoi, Thummanoon Prodpran, Soottawat Benjakul

**Affiliations:** 1 International Center of Excellence in Seafood Science and Innovation, Faculty of Agro-Industry, Prince of Songkla University, Hat Yai 90110, Songkhla, Thailand; thummanoon.p@psu.ac.th (T.P.); soottawat.b@psu.ac.th (S.B.); 2 Program of Food Science and Technology, Faculty of Agro-Industry, Prince of Songkla University, Hat Yai 90110, Songkhla, Thailand; kartoonn2547@gmail.com; 3 Center of Excellence in Bio-Based Materials and Packaging Innovation, Faculty of Agro-Industry, Prince of Songkla University, Hat Yai 90110, Songkhla, Thailand

**Keywords:** salmon skin collagen, acid-soluble collagen solution, film, rheological properties, film properties

## Abstract

Salmon skin is a byproduct from the fish processing industry that can be used as a potential source of collagen. Due to the presence of other constituents, pretreatment of the skin is required prior to the preparation of the acid-soluble collagen (ASC) solution and film. This study aimed to investigate the effects of ultrasonication amplitudes (50% and 70%) and times (5, 10, and 15 min) on the properties and characteristics of ASC solutions and films. The ASC solutions had higher elastic behavior when ultrasonication at a lower amplitude and a shorter time was used. Films from solutions ultrasonicated at 50% amplitude had a higher thickness, tensile strength, elongation at break, and water vapor barrier property than films from solutions ultrasonicated at 70% amplitude, regardless of the ultrasonication time used. A longer ultrasonication time decreased the L* value but increased the transparency value. The FTIR spectra indicated that structural modifications were affected by the ultrasonication conditions used. The SEM images showed a continuous surface for all the films. Higher amplitudes and longer times reduced the thermal stability and crystallinity, respectively, as determined by differential scanning calorimetry and thermogravimetric analysis as well as X-ray diffraction. Therefore, ultrasonication at 50% amplitude for 10 min was suitable for producing films with satisfactory mechanical and water vapor barrier properties.

## 1. Introduction

Packaging materials are widely used to protect food products from the surroundings, retard deterioration, extend shelf life, maintain quality, and distribute foods and products conveniently. Petroleum-based materials are commonly used and have a wide range of advantages [1]. However, these materials are not biodegraded easily and generate environmental pollution [2]. It is very important to move toward alternative raw materials to replace plastic. Biopolymer films have gained increasing interest in their use as biodegradable and/or edible food packaging. Food biopolymer films can be defined as a thin continuous layer of biopolymer materials that can be applied as a coating on food, used as a wrap or pouch to hold the food or to protect it against external factors [3]. Among biopolymers, proteins are heteropolymers containing a variety of amino acids, which can undergo a wide range of interactions and chemical reactions [4]. Proteins from different origins have been utilized for biodegradable film fabrication owing to their abundance, good film-forming property, and nutritive value [5]. Among proteins, collagen, which is the major structural protein of animal connective tissue, contributing to the strength and support of tissues in skin, tendons, bones, cartilage, etc., has been widely employed as potential raw material for the fabrication of active edible films [5,6].

Collagen has been widely used in food, biomedical, pharmaceutical, and cosmetic applications [4]. Commercial collagens are generally produced from bovine and porcine hides and bones. Currently, increasing attention is being paid to alternative sources for the replacement of mammalian collagens, especially from fish processing byproducts [7]. Fish collagen can be used for halal and kosher products served for Muslim and Jewish communities, respectively. Because of the outbreak of bovine spongiform encephalopathy and bird flu, there is an increasing demand for fish collagen [7,8]. Current fish processing practically generates large amounts of byproducts, accounting for up to 60% of the total fish weight [9]. Despite the presence of several valuable components, fish processing byproducts are usually dumped in landfills or into the oceans, leading to potentially harmful environmental effects. Those byproducts are also used as raw materials for fish meal, silage, and fertilizer manufacturing [10]. Typically, fish processing byproducts consist of the viscera, head, trimming, skin, scale, roe, and bone, as well as fish that are damaged or unsuitable for human consumption, known as the ‘bycatch’ [10]. Fish processing byproducts contain a wide range of nutritional components, especially protein. By the potential use of processing or technology, marketable new products with added value can be obtained through which the processors can gain increased revenue [7].

The production of collagen powder requires several tedious processes, e.g., pretreatment with alkaline and acid, acid/pepsin solubilization, filtration, precipitation, dialysis, and lyophilization, taking more time. To conquer this problem, the treatment of raw materials in acid to obtain collagen slurries or pastes before film formulation, casting, and drying has been developed [11]. The production time for the preparation of collagen can be reduced prior to film making. In general, gelatin-based films have been widely produced. However, gelatin is thermally denatured or partially degraded collagen. As a consequence, the use of native collagen in the form of a triple helix might provide strong strands for making a film network rather than a film made from gelatin. Additionally, solubilization is the prerequisite process for the preparation of film-forming solutions and films. The ultrasonication process is an advanced and promising technique for extraction and solubilization [12], which could affect the properties of FFSs and collagen films; however, little information on films from native collagen as affected by the ultrasonication process has been reported. The impacts of ultrasonication conditions on the properties and characteristics of salmon skin collagen solutions and films, therefore, were investigated in the present study.

## 2. Materials and Methods

### 2.1. Chemicals

Acetic acid and hydrochloric acid (HCl) were procured from Merck (Darmstadt, Germany). Sodium chloride and sodium hydroxide were purchased from KemAus™ (Phakanong, Bangkok, Thailand). All the chemicals in this study were of analytical grade.

### 2.2. Pretreatment of Salmon Skin

Salmon skins without remaining meat were collected from fish processing plants, frozen and transferred to the International Center of Excellence in Seafood Science and Innovation (ICE-SSI), Faculty of Agro-Industry, Prince of Songkla University, Hat Yai, Songkhla. The frozen skins were cut into small pieces (1.5 cm × 1.5 cm) and subjected to non-collagenous protein removal, swelling and defatting processes, as tailored by Nilsuwan et al. [13]. Briefly, skins were mixed with 1.5 M NaOH solution containing 0.5 M NaCl (1:10, *w*/*v*) and stirred gently for 1 h, followed by draining. Alkali pretreatment was performed in the same manner a total of three times. Alkali-treated skins were washed with water until neutral pH was achieved. The prepared skins were treated with 0.05 M citric acid (1:10, *w*/*v*) with gentle agitation for 15 min and allowed to stand for 45 min. Swollen skins were washed with water until neutral pH was reached. For fat removal from the skin, the pretreated skins were mixed with 30% (*v*/*v*) isopropanol at a skin/solvent ratio of 1:10 (*w*/*v*). The mixture was mixed with the aid of an ultrasonic processor (Sonics, Model VC750, Sonics & Materials, Inc., Newtown, CT, USA), using a 13 mm probe, at an amplitude of 70% with pulse mode for 10 min. The mixture was further stirred at 150 rpm for a total of 60 min with an overhead stirrer (RW 20 digital, IKA-Werke GmbH & CO.KG, Staufen, Germany). All the operations were carried out at refrigerated temperature (4–8 °C). Finally, the pretreated salmon skin (PSS) was collected, washed with distilled water 3 times, and packaged in polyethylene bags. The PSS contained 80.79% moisture (wet basis), 96.31% protein (dry basis), 3.56% fat (dry basis), and 0.34% ash (dry basis), as determined by the AOAC method [14].

### 2.3. Preparation of Acid-Soluble Collagen (ASC) Solution

The ASC from PSS was prepared at a protein concentration of 6% (*w*/*v*). The PSS was cut into small pieces (0.5 cm × 0.5 cm) and subjected to ultrasonication using an ultrasonic processor in acetic acid solution (pH 2.0) at amplitudes of 50% and 70% for 5, 10 and 15 min using pulse mode (5 s pulse on/5 s pulse off) under controlled temperature in an ice bath. Then, the mixtures were stirred at 4 °C for 24 h to obtain a homogenous solution. Thereafter, the slurries containing acid-soluble collagen (ASC) were adjusted to the designated final volume with the acetic acid solution (pH 2.0). All ASC solutions prepared under different conditions were used for the determination of rheological properties as well as preparation of films.

### 2.4. Rheological Properties of ASC Solutions

The oscillatory measurement of ASC solutions was conducted using a RheoStress RS1 rheometer (HAAKE, Karlsruhe, Germany) equipped with 60 mm diameter stainless-steel parallel plate. Dynamic frequency sweeps for samples were performed on the samples in the range of 0.1 to 10 Hz at 25 °C with a constant strain of 5% [15]. The storage modulus (G′), loss modulus (G″), and loss factor (tan δ) were recorded.

### 2.5. Preparation of Films

The ASC solutions (4.0 ± 0.1 g) were cast onto a rimmed silicone resin plate (5 cm × 5 cm). The samples were air-blown for 24 h at 25 ± 1 °C and subsequently dried in an environmental chamber (Model KBF 115, Binder GmbH, Tuttlingen, Germany) at 25 ± 0.5 °C with 50 ± 5% relative humidity for 48 h. The dried collagen films were manually peeled off and analyzed.

### 2.6. Analysis of Films

#### 2.6.1. Film Thickness

Thickness was measured using a digital micrometer (Mitutoyo Corp., Kawasaki-shi, Japan). Each film was measured at five random positions, and the average thickness was calculated from ten film samples.

#### 2.6.2. Mechanical Properties

Mechanical properties including tensile strength (TS), elongation at break (EAB) and Young’s modulus (YM) of the films were tested as per the method of Nilsuwan et al. [16] with slight modifications. Ten films (2 cm × 5 cm) were examined using Universal Testing Machine (Model H10KS, TINIUS OLSEN, Surrey, England) with a crosshead speed of 30 mm/min and an initial grip length of 30 mm, equipped with a tensile load cell of 200 N.

#### 2.6.3. Water Vapor Permeability (WVP)

WVP was determined following the ASTM method [17] with slight modifications as described by Nilsuwan, Arnold, Benjakul, Prodpran and de la Caba [16]. The films (4 cm × 4 cm) were placed between the airtight rubber gasket (applied with silicone vacuum grease) of aluminum permeation (gas) cups containing dehydrated silica beads (0% RH). The cups were kept in an environmental chamber (25 ± 0.5 °C and 50 ± 5% RH) and weight changes were recorded every 1 h over a period of 10 h. WVP of the film was calculated using the following equation:

(1) $$\mathrm{WVP}\;(\frac{g\;m}{m^{2}\;s\;\mathrm{Pa}})=\frac{wl}{At(P_{2}-P_{1})}$$

where *w* is the increase in weight of the aluminum cup, *l* is the thickness of the film (m), *A* is the exposed film area (m^2^), *t* is the time of weight gain (s), *P*_2_ − *P*_1_ is the vapor pressure difference across the film (1583.7 Pa at 25 °C).

#### 2.6.4. Color

L*, a* and b* values of the films were measured using a CIE colorimeter (Hunter associates laboratory, Inc., Reston, VA, USA) with a standard illuminant D65. Before the measurement of film color, the device was standardized by using black and white color standard plates. The film samples were placed on the test cell with an aperture size of 0.75 inches. The color parameters were determined by taking three readings at different locations in triplicate for each sample. The total difference of color (∆E*) was calculated using the following equation [18]:

(2) $$∆E^{*}=\sqrt{{(∆L^{*})}^{2}+{(∆a^{*})}^{2}+{(∆b^{*})}^{2}}$$

where ∆L*, ∆a* and ∆b* represent the differences value between each color parameter of the film samples and white standard color plate used as the film background (L* = 92.83, a* = −1.03, and b* = 0.29).

#### 2.6.5. Light Transmission and Transparency Value

The light transmission in the ultraviolet (UV) and visible range of film samples was measured at selected wavelengths between 200 and 800 nm using a UV–vis spectrophotometer (UV-1800, Shimadzu, Kyoto, Japan). The transparency value of the film was calculated using the following equation [19]:

(3) $$\mathrm{Transparency}\;\mathrm{value}=\frac{-\log T_{600}}{x}$$

where *T*_600_ is the fractional transmission at 600 nm and *x* is the film thickness (mm).

#### 2.6.6. Fourier Transform Infrared (FTIR) Spectroscopy

The FTIR spectra of the films were measured using ATR-FTIR (Equinox 55, Bruker, Ettlingen, Germany). The scan was performed within the range of 400–4000 cm^−1^ with a resolution of 4 cm^−1^ for 32 scans against a background.

#### 2.6.7. Scanning Electron Microscope (SEM) Image

The microstructure of film samples was visualized with a scanning electron microscope (SEM) (Hitachi, Tokyo, Japan). The instrument was operated at an acceleration voltage of 20 kV. For the cross-section sample, the films were fractured under liquid nitrogen. The surface and cross-section specimens were mounted on a bronze holder with double-side electrically conductive carbon tape, coated with gold to enhance conductivity and then examined.

#### 2.6.8. Differential Scanning Calorimetry (DSC)

Thermal properties of films were determined using a differential scanning calorimeter (PerkinElmer DSC-7, Norwalk, CT, USA). Films (3–5 mg) were hermetically sealed in aluminum pans and analyzed over the temperature range of −20 to 200 °C at a heating rate of 5 °C/min. The maximum transition temperature was determined from the endothermic peak.

#### 2.6.9. Thermogravimetric (TGA) Spectra

Films were analyzed using a thermogravimetric analyzer (TGA7, PerkinElmer, Norwalk, CT, USA) over the temperature range of 25 to 1000 °C at a heating rate of 10 °C/min. Nitrogen was used as the purge gas at a flow rate of 20 mL/min.

#### 2.6.10. X-Ray Diffraction (XRD) Pattern

XRD of films was performed using an X-ray Diffractometer (X’ Pert MPD, PHILIPS, Eindhoven, The Netherlands) operating at 40 kV and 30 mA. The radiation was generated from a Cu-Kα (λ = 1.5418 Å) source. The diffraction data were collected over a 2θ range of 2° to 50°, where θ represents the angle of incidence of the X-ray beam on the sample.

### 2.7. Statistical Analysis

All experiments were conducted in triplicate using three different sample lots. ANOVA was performed to analyze the variance of the data. The Duncan’s multiple range test [20] was used for mean comparison. The Statistical Package for Social Science (SPSS 28.0 for windows, SPSS Inc., Chicago, IL, USA) was used for statistical analysis.

## 3. Results and Discussion

### 3.1. Rheological Properties of ASC Solution

**Figure 1:**
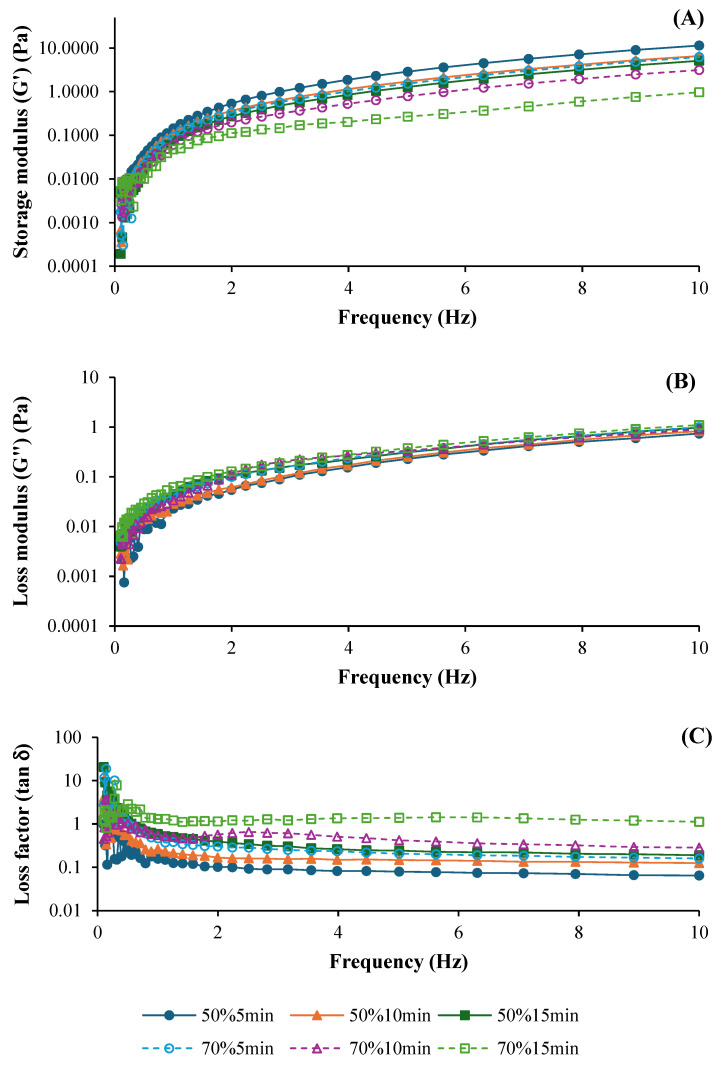
Storage modulus (G′) (**A**), loss modulus (G″) (**B**), and loss factor (tan δ) (**C**) of salmon skin collagen solutions subjected to ultrasonication at different amplitudes for various times.

The frequency sweeps performed to investigate the viscoelastic behavior of ASC solutions ultrasonicated at different amplitudes (50% and 70%) and times (5, 10, and 15 min), expressed as the elastic or storage modulus (G′), loss modulus (G″), and loss factor (tan δ), are shown in Figure 1A, 1B, and 1C, respectively. Both G′ and G″ curves were generally influenced by ultrasonication amplitude and time. The G′ curves of all solutions were increased as frequency was increased (Figure 1A), indicating predominantly elastic behavior across all treatments [21]. ASC solutions ultrasonicated at 50% amplitude had higher G′ values, compared to those treated with 70% amplitude, regardless of the ultrasonication time used. This result suggested that better elastic properties were found in ASC solutions ultrasonicated at lower amplitudes. Additionally, ASC solutions ultrasonicated for 5 min showed the highest G′, followed by those treated for 10 and 15 min, respectively. Thus, prolonged ultrasonication time could reduce the elasticity of ASC solutions, which was plausibly due to the degradation of the collagen structure to a greater degree. Ultrasound application induced a loss of fibril bundles over time because the structure became less compact [22]. Furthermore, the loss modulus (G″) also increased when the frequency increased (Figure 1B). The viscosity was slightly higher in ASC solutions ultrasonicated with shorter times (5 and 10 min) at both amplitudes, whereas the ASC solution with ultrasonication for 15 min exhibited a slight decrease in G″. The deformation of molecular entanglements and cross-links might occur with prolonged ultrasonication. Nevertheless, all ASC solutions mostly exhibited elastic behavior as tan δ < 1 (solid-like behavior) (Figure 1C) [21], indicating the elasticity of the solution. These rheological characteristics were similar to those of collagen solutions derived from albacore (*Thunnus alalunga*) [21] and calf skin [23]. Overall, all ASC solutions exhibited elastic behavior (G′ > G″), indicating strong structural integrity, particularly in treatments with lower amplitude and shorter ultrasonication times. Zheng et al. [24] also documented that collagen solutions exhibited higher G′ and G″ values than collagen/chitosan blended solutions, especially in the low-frequency range (0.01–1 Hz).

### 3.2. Properties and Characteristics of Film

#### 3.2.1. Film Thickness

The thickness of films from ASC solutions ultrasonicated with 50% and 70% amplitudes for different times is shown in Table 1. All films had a thickness in the range of 0.0280–0.0388 mm. Films formed from ASC solutions subjected to 50% amplitude generally exhibited higher thickness (*p* < 0.05), compared to those of films from solutions ultrasonicated at 70% amplitude, regardless of the ultrasonication time used. This result suggested that higher amplitude led to increased molecular breakdown and denser film packing, resulting in thinner films. This observation aligned with previous studies, which indicated that ultrasonic treatment affected collagen structure, leading to modifications in film characteristics [12,13]. At the same amplitude used, no difference in thickness of films from ASC solutions ultrasonicated with different times was observed (*p* > 0.05), except the higher thickness of the film from the solution ultrasonicated at 50% for 15 min, compared to those of films from solutions ultrasonicated for 5 and 10 min. The longer processing times (15 min) at moderate amplitude (50%) could improve collagen dispersion and cross-linking. Ultrasound treatment improved collagen film integrity and thickness due to enhanced molecular interactions and network formation [6].

#### 3.2.2. Mechanical Properties

Mechanical properties of films from salmon skin ASC solutions subjected to ultrasonication under different conditions, as expressed as tensile strength (TS), elongation at break (EAB) and Young’s modulus (YM), are shown in Table 1. Overall, films from solutions ultrasonicated at 50% amplitude exhibited higher TS than those of films from solutions ultrasonicated at 70% amplitude, regardless of the ultrasonication time used. This result suggested that a moderate amplitude (50%) was more effective for producing films with higher tensile strength, whereas excessive cavitation at 70% amplitude might cause excessive disruption of collagen molecules, leading to weaker films. Similar observations were reported in studies where excessive ultrasonic energy caused molecular degradation, reducing the strength of biopolymer-based films [25]. Furthermore, within the same amplitude level, a decreasing TS value was observed (*p* < 0.05) in the resulting film as ultrasonication time increased. This finding suggested that prolonged ultrasonication time might reduce the integrity of the film matrix, potentially due to excessive fragmentation of collagen fibrils and weakening of intermolecular interactions. Similarly, films from solutions ultrasonicated at 50% amplitude generally exhibited greater flexibility compared to those of films from ASC solutions ultrasonicated at 70% amplitude, irrespective of the ultrasonication time used. The highest EAB was observed in the film from the solution ultrasonicated for 5 min at 50% amplitude. However, EAB decreased as ultrasonication time increased. This result indicated that excessive ultrasonication caused partial denaturation of collagen, leading to reduced film elasticity [25]. In addition, no difference in EAB among films from solutions ultrasonicated at 70% amplitude for 5–15 min was found (*p* > 0.05). In contrast to TS and EAB, Young’s modulus remained stable across all ultrasonication conditions. Therefore, film stiffness was not substantially affected by ultrasonication amplitude or duration, which primarily influenced tensile strength and elongation at break. Overall, the amplitude and time of ultrasonication impacted the mechanical properties of collagen-based films. Lower amplitude was preferable for achieving flexibility and mechanical strength in the resulting film. Therefore, moderate ultrasonication amplitude (50%) and time (10 min) might be the optimal conditions for producing films with a satisfactory tensile strength, flexibility, and stiffness.

#### 3.2.3. Water Vapor Permeability (WVP)

The WVP of salmon skin collagen (SSC) films from ASC solutions subjected to various ultrasonication conditions is shown in Table 1. Films from solutions ultrasonicated at 70% amplitude exhibited higher WVP, compared to those of films from solutions ultrasonicated at 50% amplitude, particularly under longer processing times. The intense ultrasonication could disrupt the collagen network, leading to structural changes that facilitated water vapor migration through the film, thereby reducing its effectiveness as a moisture barrier. However, the result was different from Qu, Guo, Zhang, Jin, Wang, Wahia and Ma [25] who documented that the WVP of collagen–chitosan (at ratio of 1:4) composite film was lowered after ultrasonic treatment with an ultrasonic frequency of 28 kHz, power density of 100 W/L, sweep frequency cycle of 100 ms, sweep frequency amplitude of ±0.5 kHz, duty ratio of 77% (10 s/3 s) and time of 10 min. Proper ultrasound conditions promoted the interaction of collagen with other components and increased the degree of cross-linking, thereby making the film more compact. As a result, the penetration and escape of water vapor were reduced, as evidenced by reduced WVP [25]. Furthermore, lower amplitude (50%) and shorter ultrasonication time (10 min) were more suitable for producing films with low WVP. Therefore, optimal ultrasonication conditions were essential for balancing mechanical strength and barrier properties in collagen-based films.

#### 3.2.4. Color

The color parameters, including L*, a*, b* and ΔE* values of fish collagen films from solutions treated with ultrasonication at various amplitudes and times, are presented in Table 2. All salmon skin collagen-based films had L*, a*, and b* values in the value ranges of 90.22–91.20, (−2.00)–(−1.87), and 4.18–6.48, respectively. Qu et al. [26] reported that tuna skin collagen–chitosan (TSC-CTS) composite film without ultrasound and polyphenol modification had L*, a*, and b* values at 94.91, (−1.32), and 11.60, respectively. Films from solutions ultrasonicated with 70% amplitude exhibited higher L* values than those of films from solutions ultrasonicated at 50% amplitude (*p* < 0.05), suggesting that higher ultrasonication amplitudes resulted in lighter films. This effect might be attributed to more efficient dispersion of collagen molecules and a reduction in particle size, leading to a smoother and more uniform film surface. However, a decrease in L* value was observed for films from ASC solutions treated at both amplitudes as ultrasonication time increased (*p* < 0.05). This reduction in brightness could be linked to increased oxidation and molecular degradation, thus negatively affecting color retention [12]. No significant differences in a* values were found among all films from solutions ultrasonicated under different conditions (*p* > 0.05). Films from solutions ultrasonicated at 50% amplitude generally exhibited higher b* and ΔE* values, compared to those of films from solutions ultrasonicated at 70% amplitude, regardless of the ultrasonication time used. Thus, lower amplitude could preserve the natural coloration of collagen, while higher amplitude might promote structural modifications, affecting overall color.

#### 3.2.5. Light Transmission and Transparency Value

**Figure 2:**
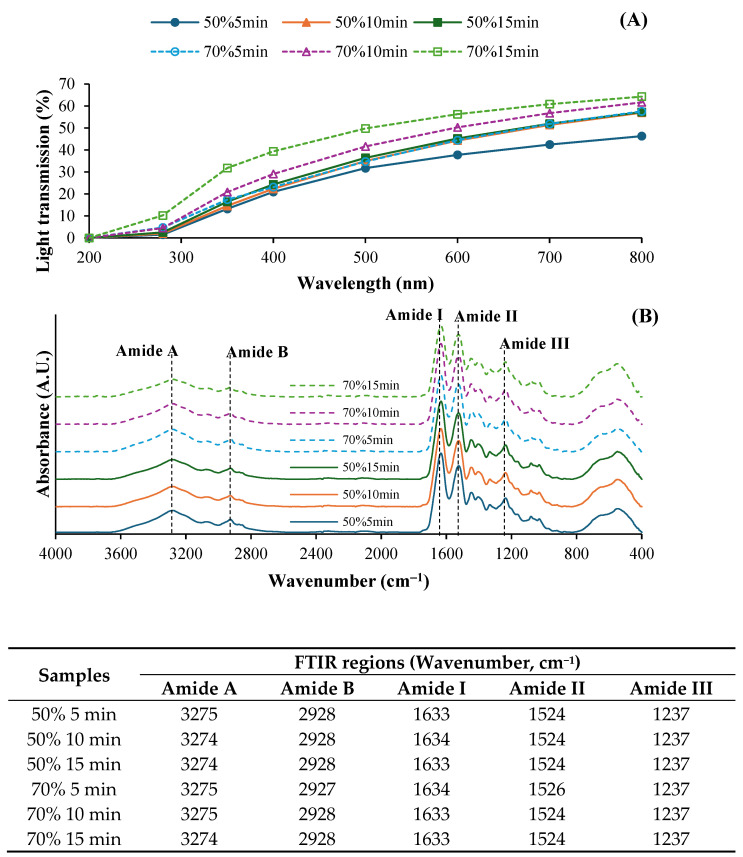
Light transmission (**A**) and Fourier transform infrared (FTIR) spectra (**B**) of films from salmon skin collagen solutions subjected to ultrasonication at different amplitudes for various times.

Light transmission and transparency values of fish collagen films from ASC solutions subjected to various ultrasonication conditions are depicted in Figure 2A and Table 2, respectively. Generally, all films exhibited high UV barrier properties in the wavelength range of 200–350 nm. However, light transmission was increased in the wavelength range of 400–800 nm, indicating that films became more transparent. Protein-based films typically have high UV barrier properties and high visible light transmission [27]. Moreover, films from ASC solutions ultrasonicated at 70% amplitude exhibited higher light transmission compared to those of films from solutions treated at 50%, regardless of the ultrasonication time used. Higher ultrasonic intensity might result in films with greater transparency, likely due to a looser film structure allowing more light to pass through. At the same amplitude, films from solutions ultrasonicated for longer times showed the increased light transmission, suggesting that prolonged ultrasonication enhanced film clarity by improving molecular dispersion. Nevertheless, the transparency value was also increased in films from solutions ultrasonicated at higher intensity (*p* < 0.05) (Table 2). Higher ultrasonication amplitude and longer time more likely enhanced molecular alignment and reduced thickness or density, leading to increased transparency values in the resulting film.

#### 3.2.6. Fourier Transform Infrared (FTIR) Spectroscopy

The FTIR spectra of SSC films from the solutions ultrasonicated under different conditions showed distinct absorption peaks corresponding to the characteristic functional groups of collagens, particularly in the amide regions (Figure 2B). All film samples exhibited similar major peaks, including Amide A, B, I, II, and III. No difference in the wavenumbers of those major peaks between all films was observed. The broad peak in the range of 3274–3275 cm^−1^ (Amide A) corresponded to N-H stretching vibrations, indicating the presence of hydrogen bonding [13]. The Amide B band, observed around 2900 cm^−1^, was associated with asymmetric CH_2_ stretching vibrations [13]. A slightly lower intensity of both peaks was generally attained for films from solutions subjected to longer ultrasonication time. This result might be associated with lower interaction between collagen via a hydrogen bond to some degree, which was induced by the prolonged ultrasonication time [12]. Moreover, the key structural bands for collagen, Amide I (1600–1700 cm^−1^), Amide II (1500–1600 cm^−1^), and Amide III (1200–1300 cm^−1^) [12], were presented in all samples. Amide I represents C=O stretching vibrations of the polypeptide backbone, indicating the secondary structure of the collagen films. The amide II band corresponded to N–H bending [12]. Films treated at 70% amplitude for 15 min exhibit lower intensity of both Amide I and II. This reduction might be related with the formation of a hydrogen bond between adjacent chains via NH groups [12]. Furthermore, the Amide II band, associated with the combination of N-H deformation and C-N stretching, was involved in the intermolecular interactions of collagen [13]. All ASC samples displayed amide III at the wavenumber of 1237 cm^−1^. The triple-helical structure of collagens was substantiated by the absorption ratio between amide III and 1450 cm^−1^. The ratio values of all collagen films were in the range of 0.82–0.91, which less than 1.0. This finding indicated the impact of ultrasonication on the loss of molecular ordered structure of collagen to some extent. Therefore, ultrasonication at lower amplitude and for a shorter time could be an appropriate condition for the preparation of acid-soluble collagen solutions, maintaining the structural integrity and molecular organization of the collagen in the film matrix.

#### 3.2.7. Scanning Electron Microscope (SEM) Images

**Figure 3:**
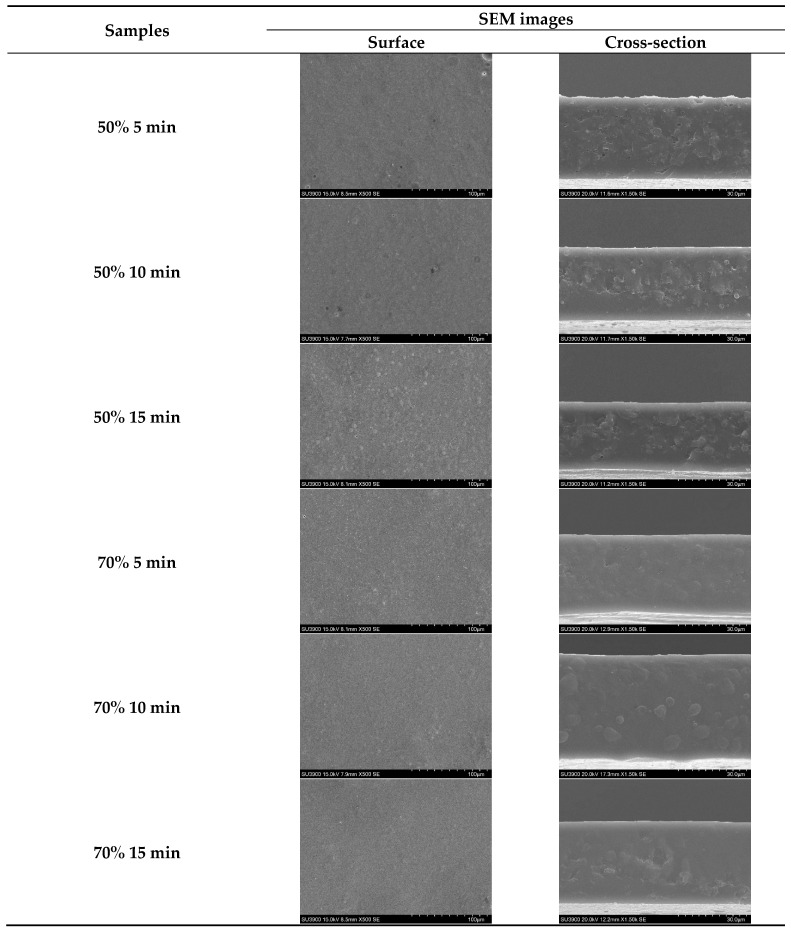
Scanning electron microscopy (SEM) images of films from salmon skin collagen solutions subjected to ultrasonication at different amplitudes for various times.

The SEM images showed the surface and cross-section morphologies of SSC films from ASC solutions treated with ultrasonication at different amplitudes for various times (Figure 3). All films exhibited a similar continuous film surface with few small pinholes. This result indicated that films from all solutions subjected to various ultrasonication conditions maintained a stable solution system with no phase separation occurring during casting and drying. For the cross-section, films from solutions ultrasonicated at 70% amplitude showed a smoother structure compared to those of films from solutions ultrasonicated at 50% amplitude, regardless of the ultrasonication time used. Protein–protein disruption in the film matrix caused by ultrasonication might contribute to the reduction in the roughness of the cross-section of the film. Higher amplitude and longer time might lead to an increase in ordered structure breakdown, making the polymer alignment closer. This more likely brought about the continuous network, as evidenced by the increased amount of smoother network. The film microstructure might be associated with film thickness and transparency value.

#### 3.2.8. Differential Scanning Calorimetry (DSC)

**Figure 4:**
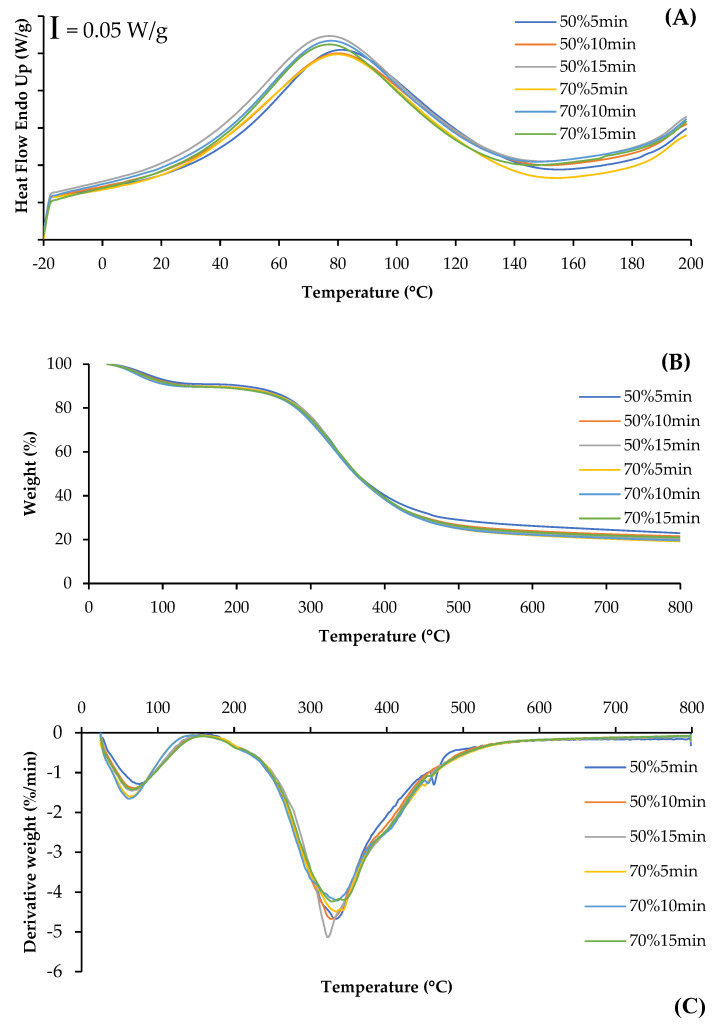
Differential scanning calorimetry (DSC) (**A**), weight loss (**B**), and derivative weight (**C**) of films from salmon skin collagen solutions subjected to ultrasonication at different amplitudes for various times.

The DSC thermogram shows the heat flow curves for SSC films from ASC solutions treated with different ultrasonication conditions (Figure 4A). The endothermic peak of all films was observed in the range of 76.25–80.67 °C, representing the thermal denaturation temperature (*T*_d_) of the collagen films, which was associated with the destruction of triple-helix structures [28]. Films from ASC solutions ultrasonicated at higher amplitude and for longer time showed a slightly lower *T*_d_, compared to those of films from solutions ultrasonicated at lower amplitude and for shorter time. This result indicated that higher energy input from ultrasonication might destroy the integrity of collagen fibers and result in lowered thermal stability of the film matrix. Shi, Liu, Yu, Chang, Goff and Zhong [28] documented that collagen films with less fiber integrity, as effected by aging treatment, exhibited lower endothermic peak temperatures. Additionally, films from ASC solutions ultrasonicated with lower amplitude and for shorter time showed a narrower and more pronounced peak, suggesting a more ordered collagen structure [29], compared to those of films from ASC solutions ultrasonicated under harsher conditions. This denaturation of triple-helix structures and ordered structures might cause a decrease in TS of collagen films, particularly at higher ultrasonication amplitude and longer times (Table 1).

#### 3.2.9. Thermogravimetric (TGA) Spectra

The TGA and derivative thermogravimetric (DTG) thermograms illustrate the thermal degradation behavior of collagen films from ASC solutions subjected to ultrasonication under various conditions (Figure 4B,C). Generally, all film samples presented similar behavior with three weight loss stages. The first stage of weight loss was observed approximately at the temperature range of 62.6–76.0 °C, mainly related to the loss of free and absorbed water. The second stage of weight loss at temperatures around 200 °C was most likely associated with the loss of low-molecular-weight protein fractions. Additionally, the third stage of weight loss was observed at 322–336 °C for all films and was related to the degradation of larger size or highly associated collagen fibers. Shi, Liu, Yu, Chang, Goff and Zhong [28] also reported that three main weight loss stages in the ranges of 50–130 °C, 190–300 °C and 300–350 °C were, respectively, observed for collagen films without or with aging treatment. Lower weight loss and derivative weight of the first and third stages were generally observed in the films from ASC solutions ultrasonicated at lower amplitude and for shorter time, compared to those of films from ASC solutions ultrasonicated at higher amplitude and for longer time. This was mostly due to the higher collagen integrity and stronger interactions between the components in ASC solution ultrasonicated under mild conditions, leading to a greater heat resistance in the resulting films. Therefore, ultrasonication under mild conditions could yield the resulting collagen film with high thermal stability.

#### 3.2.10. X-Ray Diffraction (XRD) Patterns

**Figure 5:**
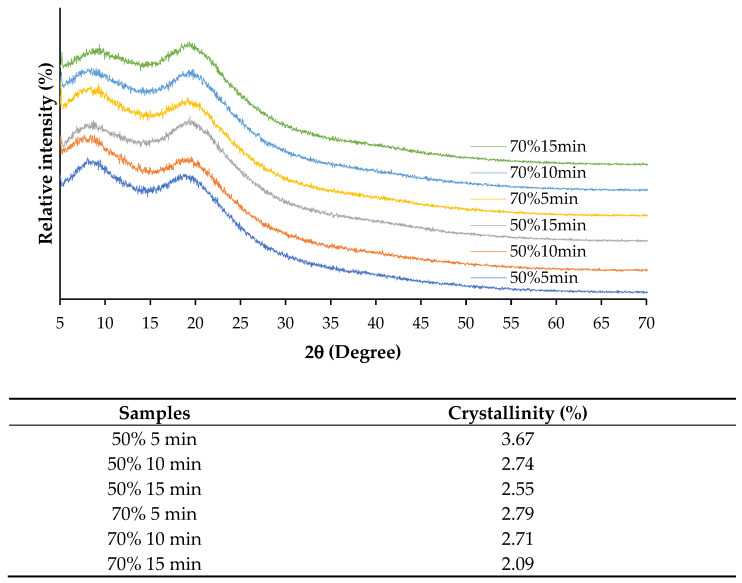
X-ray diffraction pattern and crystallinity of films from salmon skin collagen solutions subjected to ultrasonication at different amplitudes for various times.

Figure 5 shows XRD patterns and crystallinity of SSC films from ASC solutions subjected to different ultrasonication amplitudes for various times. XRD patterns can indicate the types of collagen structure, particularly the collagen-like triple-helical structure content [28]. All films exhibited two major peaks with differences in intensity at approximately 7° (2θ) and 20° (2θ). Films from ASC solutions ultrasonicated at lower amplitude for shorter time showed higher intensity of the first peak with lower intensity of the second peak, compared to those of films from ASC solutions ultrasonicated at higher amplitude and for longer time. The first peak at 7.5° (2θ) represents the content of the triple helix, while the second peak at 15–22.5° (2θ) represents the content of the amorphous phase [28,30]. This result indicated that films from ASC solutions ultrasonicated at lower amplitude for shorter time retained higher amounts of triple-helix structure than those of films from ASC solutions subjected to higher ultrasonication conditions. Moreover, films from ASC solutions ultrasonicated at lower amplitude for shorter time also exhibited higher crystallinity (Table 2) than those of films from ASC solutions ultrasonicated under harsher conditions, suggesting that lower energy input could preserve the ordered structure of the collagen matrix. The decline in crystallinity under higher ultrasonication conditions correlated with mechanical properties, as films with lower crystallinity tended to have reduced tensile strength but increased flexibility. Overall, the findings confirmed that ultrasonication significantly influenced the structural organization of collagen films, where moderate amplitude (50%) and shorter processing times (5 min) were more effective in maintaining crystallinity, while higher amplitude and prolonged exposure led to greater structural disruption.

## 4. Conclusions

The ultrasonication process influenced the properties and characteristics of salmon skin collagen solutions and the resulting films. Higher ultrasonication amplitude and time induced structural modifications, as indicated by rheological properties. Increasing ultrasonication amplitude and time generally reduced tensile strength and elongation at break as well as augmenting water vapor permeability. Higher amplitude and longer time also provided greater film transparency with some changes in color parameters. The films had a continuous surface when lower amplitude was applied. The film from ASC solution ultrasonicated with a mild ultrasonication process had high thermal stability. The reduction in crystallinity was obtained when a high intensity of ultrasonication was employed. Therefore, films from ASC solution subjected to ultrasonication at 50% amplitude for 10 min exhibited satisfactory thickness (0.033 mm), light yellow color, slight opacity, and high tensile strength and elongation at break as well as high water vapor/UV light barrier properties and collagen matrix integrity, making them suitable for use as food packaging.

## Figures and Tables

**Table 1:** Film thickness, mechanical properties, and water vapor permeability of films from salmon skin collagen solutions subjected to ultrasonication at different amplitudes for various times.

Ultrasonication Conditions		Film Thickness	TS	EAB	YM	WVP
Amplitudes (%)	Times (min)	(mm)	(MPa)	(%)	(MPa)	(×10^−11^ g mm/m^2^ s Pa)
50	5	0.035 ± 0.003 * b	35.69 ± 1.78 a	2.73 ± 0.21 a	3400 ± 241 a	4.67 ± 0.37 c
	10	0.033 ± 0.003 b	33.34 ± 3.52 ab	2.33 ± 0.39 ab	3393 ± 204 a	4.75 ± 0.17 c
	15	0.039 ± 0.004 a	31.44 ± 2.03 b	2.21 ± 0.43 b	3108 ± 342 a	4.89 ± 0.14 bc
70	5	0.029 ± 0.004 c	30.28 ± 3.35 b	2.26 ± 0.37 b	3164 ± 445 a	4.96 ± 0.49 bc
	10	0.028 ± 0.004 c	25.49 ± 4.78 c	2.07 ± 0.16 b	3066 ± 558 a	5.38 ± 0.40 ab
	15	0.029 ± 0.003 c	24.38 ± 3.42 c	2.00 ± 0.34 b	3240 ± 536 a	5.77 ± 0.38 a

* Values are mean ± SD (*n* = 3). TS: tensile strength; EAB: elongation at break; YM: Young’s modulus; WVP: water vapor permeability. Different lowercase letters in the same column indicate significant differences (*p* < 0.05).

**Table 2:** Color and transparency value of films from salmon skin collagen solutions subjected to ultrasonication at different amplitudes for various times.

Ultrasonication Conditions		L* Value	a* Value	b* Value	ΔE* Value	Transparency Value
Amplitudes (%)	Times (min)					
50	5	90.82 ± 0.08 * b	−1.94 ± 0.06 a	5.41 ± 0.19 bc	5.60 ± 0.20 bc	11.16 ± 0.42 f
	10	90.60 ± 0.07 c	−2.00 ± 0.07 a	5.88 ± 0.48 b	6.19 ± 0.74 b	11.65 ± 0.19 e
	15	90.22 ± 0.12 d	−1.88 ± 0.08 a	6.48 ± 0.37 a	6.77 ± 0.37 a	13.39 ± 0.25 d
70	5	91.20 ± 0.16 a	−1.87 ± 0.03 a	4.18 ± 0.34 d	4.44 ± 0.35 d	15.52 ± 0.36 c
	10	90.87 ± 0.12 b	−1.87 ± 0.05 a	5.17 ± 0.41 c	5.31 ± 0.49 c	17.90 ± 0.04 b
	15	90.58 ± 0.13 c	−1.90 ± 0.09 a	5.68 ± 0.88 bc	6.08 ± 0.93 b	19.45 ± 0.44 a

* Values are mean ± SD (*n* = 3). Different lowercase letters in the same column indicate significant differences (*p* < 0.05).

## Data Availability

The original contributions presented in the study are included in the article, further inquiries can be directed to the corresponding author.
